# PINK1 and Parkin – mitochondrial interplay between phosphorylation and ubiquitylation in Parkinson's disease

**DOI:** 10.1111/febs.13127

**Published:** 2014-11-20

**Authors:** Agne Kazlauskaite, Miratul M K Muqit

**Affiliations:** 1 MRC Protein Phosphorylation and Ubiquitylation Unit, University of DundeeUK; 2 College of Medicine, Dentistry & Nursing, University of DundeeUK

**Keywords:** kinase, mitochondria, PINK1, parkin, Parkinson's disease, ubiquitin

## Abstract

The discovery of mutations in genes encoding protein kinase PTEN-induced kinase 1 (PINK1) and E3 ubiquitin ligase Parkin in familial Parkinson's disease and their association with mitochondria provides compelling evidence that mitochondrial dysfunction is a major contributor to neurodegeneration in Parkinson's disease. In recent years, tremendous progress has been made in the understanding of how PINK1 and Parkin enzymes are regulated and how they influence downstream mitochondrial signalling processes. We provide a critical overview of the key advances in the field and also discuss the outstanding questions, including novel ways in which this knowledge could be exploited to develop therapies against Parkinson's disease.

## Introduction

As we approach two centuries since the first description of Parkinson's disease (PD), recent progress has started to reveal the origins and mechanisms of this progressive movement disorder. A leading hypothesis that has emerged over the last 30 years is that mitochondrial dysfunction may underlie the pathogenesis of PD [1]. Initial evidence arose from the observation that accidental exposure to the neurotoxin 1-methyl-4-phenyl-1,2,3,6-tetrahydropyridine (MPTP) led to Parkinsonism in humans [2]. 1-Methyl-4-phenyl-1,2,3,6-tetrahydropyridine is a selective inhibitor of complex I of the electron transport chain [3] and the relevance of this finding to sporadic PD was suggested by post-mortem studies of PD patient brains revealing reduced complex I activity in the substantia nigra, which is the major site of pathology [4,5]. Furthermore, mitochondrial dysfunction was shown to increase in the brain during normal ageing, providing an explanation for the late age of onset of PD cases [6]. Nevertheless, outstanding questions relating to the contribution of mitochondrial impairment remain, especially regarding whether it has a primary or secondary role in PD pathogenesis.

Over recent decades, PD research has been revolutionized by the identification of gene mutations in rare familial forms of PD [7]. In particular, the discovery of mutations in PTEN-induced kinase 1 (PINK1) and Parkin [8,9] and their subsequent analysis have repositioned mitochondrial dysfunction back at the heart of PD. It has also raised significant interest in the field with respect to uncovering the physiological roles of these PD-linked proteins in mitochondrial signalling and understanding how mutations lead to neurodegeneration. In this mini-review, we discuss the key advances in our understanding of the regulation and downstream signalling of PINK1 and Parkin and highlight outstanding questions for future research.

## PINK1: sensor of mitochondrial damage

Undoubtedly, the discovery of mutations in PINK1, first reported 10 years ago in patients with early-onset PD, represents a landmark finding because it provided the first direct evidence for mitochondrial dysfunction playing a primary role in the development of PD [8]. Protein kinases epitomize classical signal transduction molecules that sense and integrate external stimuli to generate changes in enzyme activity, localization or stability, which subsequently leads to the dramatic alteration of downstream signalling events and cell fate [10]. Uncovering the molecular function of protein kinases has led to profound insights into the molecular mechanisms of human diseases, particularly cancer, and has resulted in the development of numerous targeted therapies now in clinical use [11]. As such, there has been significant interest in the PD field aiming to determine the function of PINK1.

**Figure 1:**
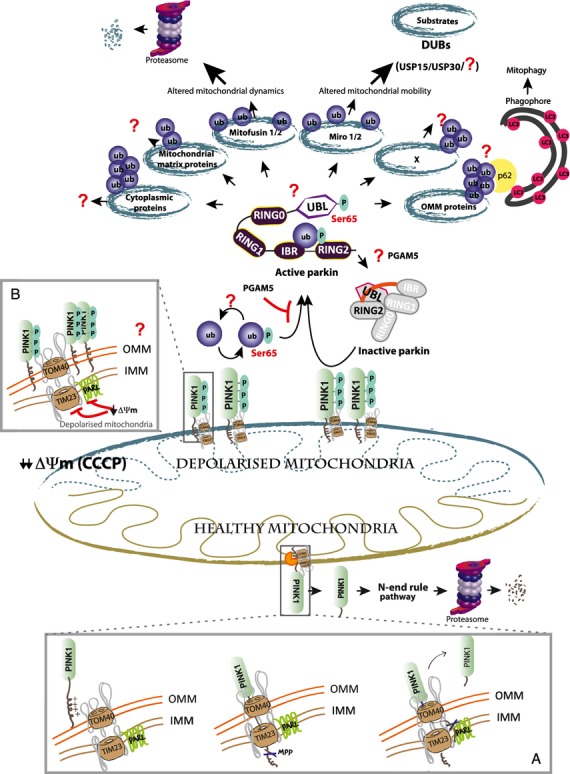
PINK1-Parkin signalling. Under healthy conditions, PINK1 is imported to the mitochondria via a multisubunit complex, including TOM40 on the outer mitochondrial membrane (OMM) and TIM23 in the inner mitochondrial membrane (IMM). Upon entry, PINK1 is sequentially cleaved by proteases MPP and PARL between residues 103 and 104. The resulting C-terminal fragment is rapidly degraded via the N-end rule pathway (inset A). Upon mitochondrial depolarization (e.g. induced by uncouplers), mitochondrial import and cleavage by PARL is inhibited, resulting in PINK1 stabilization and accumulation at the OMM, which in turn leads to PINK1 autophosphorylation and activation (inset B). Parkin exists in an inactive state mediated by multiple autoinhibitory surface interactions. Upon activation, PINK1 phosphorylates Parkin at Ser65 within its Ubl domain and ubiquitin at Ser65. Phosphorylation of Parkin Ubl Ser65 and binding of ubiquitin Ser65 confers maximal activation of Parkin E3 ubiquitin ligase activity. Multiple Parkin substrates have been identified, implicating its role in distinct mitochondrial signalling processes, although only a few substrates have been characterized in detail. Parkin-dependent ubiquitylated OMM substrates interact with ubiquitin binding domain-containing proteins (UBD) e.g. p62 that stimulate recruitment of autophagy machinery to induce mitophagy. Targeting of mitochondrial GTPases Miro1 and Mfn1/2 may influence mitochondrial transport and dynamics, respectively. The negative regulators of this pathway remain largely unknown, although recent work has suggested roles for USP15 and USP30 as deubiquitylases.

PINK1 encodes a Ser/Thr protein kinase that is unique amongst all kinases as a result of the presence of a N-terminal mitochondrial targeting domain and three insertional loops within its catalytic kinase domain. Under basal conditions, PINK1 is imported into mitochondria via the TOM 40 and TIM 23 core-containing complexes [12], where it then undergoes sequential cleavage by mitochondrial proteases [mitochondrial processing peptidase (MPP) and presenilin-associated rhomboid-like protein (PARL)] [13–16] (Fig.1). The generation of cleaved PINK1 triggers its rapid degradation via the N-end rule pathway [17] thereby maintaining low levels of PINK1 in healthy cells. Strikingly, upon treatment with mitochondrial uncouplers that induce mitochondrial depolarization [e.g. carbonyl cyanide *m*-chlorophenyl hydrazone (CCCP)], PINK1 levels were observed to stabilize by inhibition of both mitochondrial import and cleavage [18]. This led to PINK1 accumulation on the outer mitochondrial membrane (OMM) [18], where its kinase domain is exposed outwards and potentially accessible to cytosolic substrates [19]. The stabilization of PINK1 was found to be associated with an increase in its catalytic activity, as indicated directly by assessment of autophosphorylation and substrate phosphorylation [20,21] and indirectly via its ability to stimulate Parkin recruitment to damaged mitochondria [22–25].

The molecular details responsible for the sensing of mitochondrial damage and subsequent activation of PINK1 remain unclear. One possibility is that PINK1 accumulation at the OMM *per se* is sufficient to enable PINK1 to be active because it would lead to its C-terminal kinase domain becoming accessible to cytoplasmic substrates. In support of this, knockdown of MPP led to accumulation of full-length PINK1 at the OMM by inhibiting import and PINK1 was observed to be active as indicated by Parkin recruitment and mitophagy induction [16]. Furthermore, artificially tethering PINK1 to the OMM via fusion with the OPA3 mitochondrial targeting sequence caused PINK1 to be stabilized at the mitochondria and to recruit Parkin independent of mitochondrial uncouplers [22]. Fascinatingly, this is also illustrated by Parkin recruitment in the absence of mitochondrial depolarization when PINK1 is artificially tethered to non-mitochondrial membranes (e.g. the peroxisome) [12].

Another possibility is that PINK1 may undergo a conformational change upon mitochondrial depolarization that folds it into an active conformation. This has been suggested by the observation that PINK1 is activated by autophosphorylation of residues Ser228 and Ser402 [21]. The latter site, Ser402, is particularly interesting because this residue is in the putative T-loop site. A multitude of protein kinases share a common mechanism of activation by T-loop phosphorylation (achieved either through autophosphorylation or by phosphorylation by an upstream kinase) that leads to a structural alteration in the activation segment of the kinase [26]. In future studies, it would be interesting to dissect the role of these PINK1 autophosphorylation sites *in vivo* via generation of phosphospecific antibodies or by quantitative mass spectrometry (MS).

If the location of PINK1 is central to both sensing mitochondrial damage and its ensuing activation, then it will be critical to determine the localization of endogenous PINK1 under both healthy conditions and upon mitochondrial depolarization because our current understanding is based almost exclusively on the localization of over-expressed PINK1. In addition, given the intimate association between mitochondrial depolarization and mitochondrial import block, it remains a challenge to determine whether either or both are required for PINK1 activation. Further complexity in understanding how PINK1 is activated has been derived from an elegant analysis of PINK1 knockout neurones that revealed an important role for PINK1 in maintaining calcium homeostasis [27]. Because mitochondrial depolarization leads to an impaired calcium buffering capacity of mitochondria [28], it will be important to probe the potential role of calcium-dependent signalling on PINK1 activation.

Finally, although mitochondrial uncouplers have proven to be extremely useful tools for activating PINK1 in cells and uncovering its function, the physiological equivalent stimulus in the brain remains elusive. Because ageing is a major risk factor of PD, it would be interesting to invetigate whether neurones become less efficient at maintaining mitochondrial membrane potential with age and whether this is associated with increased PINK1 catalytic activity.

## Parkin activation: dependence on PINK1

Mutations in Parkin are the major cause of familial early onset PD, accounting for almost 50% of all cases in patients under the age of 40 years [29]. Parkin is a RING-in-between-RING (RBR) E3 ligase, capable of mediating mono, multi-mono and polyubiquitylation of substrates with different chain topologies [30,31]. Historically, Parkin was assumed to be a typical RING E3 ligase, acting as a scaffold that mediates interaction between a cognate E2 and a substrate. In a landmark study, it was revealed that Parkin, as well as other RBR enzymes, possesses a catalytic cysteine within its RING2 domain (Cys431) that acts as an intermediate ubiquitin acceptor between the E2 and substrate [32]. This led to Parkin and other RBRs to be reclassified as RING/HECT hybrid E3 ligases [32]. A further metamorphosis in our understanding of Parkin was achieved when it was discovered that full-length untagged Parkin was catalytically inactive and it was proposed that the autoinhibition was mediated in part by the N-terminal ubiquitin-like domain (Ubl) [33]. Structural analysis of N-terminal deleted fragments of Parkin have confirmed that Parkin is autoinhibited and also identified two further regions of autoinhibition mediated through interactions between the RING0 and catalytic cysteine, Cys431, within the RING2 domain and blockade of the E2 binding site on the RING1 domain by a repressor element of Parkin (REP) α-helix [34–36]. However, the mechanism of Parkin activation was not revealed by these structures [34–36].

Clinically, patients harbouring PINK1 mutations resemble those with mutations in Parkin [37]. The decisive advance linking these two genes together was the discovery in *Drosophila* that PINK1^−/−^ and Parkin^−/−^ mutant flies exhibited similar mitochondrial abnormalities, as well as neuronal loss and motor deficits [38,39]. Furthermore, it was demonstrated that over-expression of Parkin could rescue the PINK1^−/−^ phenotype but not vice versa, indicating that PINK1 functions upstream of Parkin in a common mitochondrial pathway [38,39]. This work supported an earlier study in mammalian cells that found Parkin to be neuroprotective against mitochondrial damage [40].

Although Parkin is predominantly cytoplasmic, groundbreaking work revealed that Parkin could be selectively recruited to damaged mitochondria upon depolarization induced by uncouplers, such as CCCP, and that, remarkably, this stimulated their autophagic removal termed mitophagy [41]. It was further shown that Parkin recruitment was dependent on stabilization and accumulation of PINK1 on the OMM of depolarized mitochondria [22–25]. However, biochemical mechanisms linking PINK1 to its regulation of Parkin initially remained elusive as a result of the low catalytic activity of mammalian PINK1. This was overcome by the discovery of catalytically active insect orthologues of PINK1 (including *Tribolium castaneum* PINK1) that enabled the development of robust assays of PINK1 kinase activity [42]. Deployment of *Tribolium castaneum* PINK1 in a substrate screen subsequently led to the identification of Parkin as a direct PINK1 substrate and the site of phosphorylation was mapped to Ser65 that lies within the Ubl domain of Parkin [20]. Furthermore, phosphorylation of Parkin Ser65 was found to activate Parkin and stimulate E3 ligase activity [20]. However, the low resolution structure of full-length Parkin [35] reveals that the Ser65 residue lies away from the autoinhibitory interface, providing no clear explanation as to how phosphorylation at this site mediates Parkin activation. Moreover, mutation of the Parkin Ser65 site to Ala only partially prevented its translocation to depolarized mitochondria in cells, suggesting that additional regulatory factors were required for optimal Parkin activation by PINK1 [43]. Recently, in a series of three independent reports, PINK1 has been discovered to directly phosphorylate ubiquitin at residue Ser65 (equivalent to the Parkin Ubl site) and that this is required for optimal activation of Parkin E3 ligase activity [44–46] (Fig.1). The discovery of phospho-ubiquitin represents the missing link in Parkin activation by PINK1; it is also the first example of a functional phosphorylation site on ubiquitin and the most dramatic example of cross-talk between these two major forms of post-translational modifications [44–46].

The mechanisms underlying phospho-ubiquitin-mediated Parkin activation remain unknown. First, it will be important to understand whether the activation is mediated by conformational changes induced by phospho-ubiquitin binding to Parkin or by substrate preference itself. Initial reports suggest that binding of phospho-ubiquitin plays the major role in activation because a C-terminal di-glycine mutant of ubiquitin that cannot be attached to chains was found to retain the ability to activate Parkin [46].

The crystal structures of Parkin have revealed a sulphate-containing pocket within the RING0 domain of Parkin flanked by residues K161/R163/K211 that could putatively bind a phosphopeptide and act as a docking-site for phospho-ubiquitin; it would be interesting to co-crystalize phospho-ubiquitin bound to Parkin and determine whether these residues form the binding site. This structure may also provide insights into how phospho-ubiquitin renders Parkin in an active conformation. Phospho-ubiquitin is also incorporated into polyubiquitin chains, suggesting that it might have additional roles beyond Parkin activation [46]. As of yet, it is not known whether PINK1 can phosphorylate ubiquitin that is incorporated into chains and it will also be interesting to explore whether the phosphorylation is influenced by the chain topology and influences the preference for chain formation by Parkin. It would also be fascinating to investigate whether phospho-ubiquitin can confer substrate specificity for Parkin (as well as other E3 ligases) and hence enable Parkin to regulate distinct downstream signalling events. Although biochemical and cell biological analysis does support PINK1 regulation of Parkin and ubiquitin via phosphorylation at Ser65, it will be crucial to confirm that these signalling events occur *in vivo* where PINK1 and Parkin are expressed at endogenous levels. Further exploration of native cells will demand the development of tools, such as highly-sensitive antibodies, with potential utility as biomarkers of PINK1/Parkin pathway activity and that enable the quantitative monitoring of this signalling pathway in PD patients.

## Parkin: effector of mitochondrial damage

Although much progress has been made into understanding how PINK1 and Parkin are regulated, a major question remains on the cellular consequences of PINK1-directed Parkin activation after mitochondrial depolarization. To date, the field has been significantly influenced by the discovery that activation of Parkin initiates mitophagy [41]. As such, there is great interest in solving the mechanisms of Parkin-induced mitophagy and, in particular, determining the identity of the key substrates whose ubiquitylation by Parkin stimulates the recruitment of the autophagy machinery to sites of mitochondrial damage. Multiple substrates of Parkin have been previously identified upon mitochondrial depolarization [47,48]; however, only a select few have been thoroughly validated. The evidence obtained to date suggests that Parkin is a promiscuous ubiquitin ligase targeting multiple substrates at the OMM and that this collective ubiquitylation signals recruitment of the autophagosome machinery to initiate mitophagy [48]. However, it is still unknown whether specific substrates are required to drive mitophagy and, although K11, K27, K48, and K63 ubiquitin linkages have been identified in depolarized cells, it is still unknown which linkage type is critical for mitophagy. Furthermore, it is still not clear how these ubiquitin linkages are decoded. It would interesting to identify all of the ubiquitin binding domain-containing proteins that translocate to mitochondria after depolarization and also assess their role in mitophagy initiation.

Although Parkin activation drives mitophagy, it is still not known whether this occurs at endogenous levels of Parkin because the current assays to monitor mitophagy lack sensitivity and require the over-expression of Parkin to achieve robust clearance of mitochondria. The lack of sensitive assays has also prevented the assessment of whether mitophagy is altered in PD-derived tissues and cell lines. A recent fluorescence-based assay of mitophagy has been developed that enables the monitoring of mitophagy under endogenous PINK1 and Parkin levels [49]. Interestingly, both PINK1 and Parkin were found to be dispensable for mitochondrial depolarization-induced mitophagy under these assay conditions, suggesting redundancy [49]. However, that study employed small interfering RNA knockdown technologies that did not completely abolish PINK1 and Parkin levels and so it would be important to analyze this in PINK1 and Parkin knockout cells. Recently, it has been suggested that PINK1 and Parkin could regulate an autophagy-independent mitochondrial quality control pathway via the generation of vesicles enabling the removal of damaged and oxidized proteins [50]. This has revealed interesting cross-talk between the mitochondria and membrane trafficking pathways and it will be interesting to identify the molecular check-points that determine the outcome of mitochondrial damage.

The involvement of other mitochondrial processes has been implicated through identification of some well characterized Parkin substrates. Mitofusin (Mfn)1 and Mfn2 are major regulators of mitochondrial fusion and dynamics and their ubiquitylation by Parkin has been shown in multiple studies [47]. Although numerous Mfn1 and Mfn2 ubiquitylation sites have been mapped by MS [47], the key sites induced by Parkin after CCCP that promote its degradation or, alternatively, alter its function, such as its GTPase activity, remain unknown. In the future, a detailed structure–function analysis of these sites and an analysis of the impact of mutations on endogenous protein functioning would allow a more thorough understanding of the interplay between Mfn ubiquitylation and its downstream effects. Furthermore, the mechanism of how ubiquitylation of Mfn1/2 is linked to the induction of mitophagy remains to be determined.

Similarly, multiple ubiquitylation sites have been identified on the mitochondrial GTPase Miro1 [47,51]. Miro1 plays a major role in mitochondrial transport and tethers mitochondria to microtubules via the kinesin motor protein [52]. Inactivation or loss of Miro1 might lead to the detachment of kinesin and promote pools of stationary mitochondria that could be selectively degraded. However, it still is unknown whether PINK1 and Parkin regulate mitochondrial transport at the endogenous level and, moreover, which of the identified ubiquitylation sites are critical. There is also great interest in understanding the effect of Parkin ubiquitylation on other substrates, including cytoplasmic proteins and nuclear proteins. Do these events represent distinct roles of Parkin independent of its mitochondrial ubiquitome or is there likely to be cross-talk between mitochondrial damage and aberrant signalling in these other cellular compartments?

## Therapeutic implications

The discovery of phospho-ubiquitin mediated Parkin activation provides a platform for the development of small molecule activators of Parkin that mimic phospho-ubiquitin as potential therapeutic compounds. Greater knowledge of the relevant mechanism and structural insights into how phospho-ubiquitin binds will greatly aid this effort. Nevertheless, traditionally, the development of activators has proven extremely challenging in drug discovery.

Phosphorylation and ubiquitylation are reversible signalling events and there is now significant interest in uncovering the negative regulators of the pathway because these could prove to be more tractable drug targets. A start has already been made on the deubiquitylation of Parkin-directed substrates through the identification of USP15 and USP30 that are both members of the ubiquitin specific protease family of deubuitinases [53,54]. It was recently shown that knockdown of USP15 enhanced Parkin-dependent mitophagy and global upregulation of mitochondrial ubiquitylation upon CCCP [53]. However, it remains to be shown whether all or a subset of the substrates are specifically upregulated. Furthermore, USP15 does not normally reside in mitochondria and it would be crucial to determine how the recruitment and targeting of mitochondrial substrates is achieved. By contrast, USP30, a mitochondrial localized DUB, was also shown to regulate Parkin-induced mitophagy in cells including neurones [54]. The authors demonstrated that TOM20 and Miro1 (previously characterized substrates of Parkin) ubiquitylation is enhanced upon USP30 knockdown [54]. Strikingly USP30 knockdown *in vivo* could rescue the PINK1 or Parkin^−/−^ phenotypes in *Drosophila*, indicating that the inhibition of USP30 could be therapeutically advantageous in patients with equivalent null mutations in these genes [54].

## Closing thoughts

The desire to better understand PINK1 and Parkin has led to many exciting discoveries in recent years. Molecular elaboration of how PINK1 and Parkin are linked will provide a solid platform for the development of rational therapies for PD. However, a question remains: are there additional roles for PINK1 beyond Parkin regulation that could lead to further ideas about how to potentially intervene in PD? In the future, it will be exciting to discover novel PINK1 substrates and investigate their roles in PD. Their existence is suggested by a recent analysis of PINK1 knockout rats that revealed a striking neurodegeneration phenotype in direct contrast to Parkin knockout rats that did not [55]. Furthermore, PINK1 was originally found to be regulated by the tumour suppressor PTEN that regulates major signal transduction pathways in cancer and metabolism. Until recently, very little work had been undertaken on the role of PTEN in PINK1 signalling, although new data suggest a convergence of the PTEN pathway and mitochondrial dysfunction [56]. Complementing previous work on Parkin and cancer [57], recent studies have now linked PINK1 with both cancer and metabolism [58,59]. It will be fascinating to investigate the link between PTEN and PINK1 further, as well as understand the mechanism of PINK1 and Parkin disruption in other human diseases.

## Abbreviations

CCCP: carbonyl cyanide *m*-chlorophenyl hydrazone
Mfn: mitofusin
MPP: mitochondrial processing peptidase
OMM: outer mitochondrial membrane
PARL: presenilin-associated rhomboid-like protein
PD: Parkinson's disease
PINK1: PTEN-induced kinase 1
RBR: RING-in-between-RING
Ubl: ubiquitin-like
